# Interactive Multi-Robot Painting Through Colored Motion Trails

**DOI:** 10.3389/frobt.2020.580415

**Published:** 2020-10-14

**Authors:** María Santos, Gennaro Notomista, Siddharth Mayya, Magnus Egerstedt

**Affiliations:** ^1^School of Electrical and Computer Engineering, Institute for Robotics and Intelligent Machines, Georgia Institute of Technology, Atlanta, GA, United States; ^2^School of Mechanical Engineering, Institute for Robotics and Intelligent Machines, Georgia Institute of Technology, Atlanta, GA, United States; ^3^GRASP Laboratory, University of Pennsylvania, Philadelphia, PA, United States

**Keywords:** interactive robotic art, robotic swarm, painting, human-swarm interaction, heterogeneous multi-robot teams

## Abstract

In this paper, we present a robotic painting system whereby a team of mobile robots equipped with different color paints create pictorial compositions by leaving trails of color as they move throughout a canvas. We envision this system to be used by an external user who can control the concentration of different colors over the painting by specifying density maps associated with the desired colors over the painting domain, which may vary over time. The robots distribute themselves according to such color densities by means of a heterogeneous distributed coverage control paradigm, whereby only those robots equipped with the appropriate paint will track the corresponding color density function. The painting composition therefore arises as the integration of the motion trajectories of the robots, which lay paint as they move throughout the canvas tracking the color density functions. The proposed interactive painting system is evaluated on a team of mobile robots. Different experimental setups in terms of paint capabilities given to the robots highlight the effects and benefits of considering heterogeneous teams when the painting resources are limited.

## 1. Introduction

The intersection of robots and arts has become an active object of study as both researchers and artists push the boundaries of the traditional conceptions of different forms of art by making robotic agents dance (Nakazawa et al., 2002; LaViers et al., 2014; Bi et al., 2018), create music (Hoffman and Weinberg, 2010), support stage performances (Ackerman, 2014), create paintings (Lindemeier et al., 2013; Tresset and Leymarie, 2013), or become art exhibits by themselves (Dean et al., 2008; Dunstan et al., 2016; Jochum and Goldberg, 2016; Vlachos et al., 2018). On a smaller scale, the artistic possibilities of robotic swarms have also been explored in the context of choreographed movements to music (Ackerman, 2014; Alonso-Mora et al., 2014; Schoellig et al., 2014), emotionally expressive motions (Dietz et al., 2017; Levillain et al., 2018; St.-Onge et al., 2019; Santos and Egerstedt, 2020), or interactive music generation based on the interactions between agents (Albin et al., 2012), among others.

In the context of robotic painting, the focus has been primarily on robotic arms capable of rendering input images according to some aesthetic specifications (Lindemeier et al., 2013; Scalera et al., 2019), or even reproducing scenes from the robot's surroundings—e.g., portraits (Tresset and Leymarie, 2013) or inanimated objects (Kudoh et al., 2009). The production of abstract paintings with similar robotic arm setups remains mostly unexplored, with some exceptions (Schubert, 2017). While the idea of swarm painting has been substantially investigated in the context of computer generated paintings, where virtual painting agents move inspired by ant behaviors (Aupetit et al., 2003; Greenfield, 2005; Urbano, 2005), the creation of paintings with embodied robotic swarms is lacking. Furthermore, in the existing instances of robotic swarm painting, the generation paradigm is analogous to those employed in simulation: the painting emerges as a result of the agents movement according to some behavioral, preprogrammed controllers (Moura and Ramos, 2002; Moura, 2016). The robotic swarm thus acts in a completely autonomous fashion once deployed, which prevents any interactive influence of the human artist once the creation process has begun. Even in such cases where the human artist participates in the creation of the painting along with the multi-robot system (Chung, 2018), the role of the human artist has been limited to that of a co-creator of the work of art, since they can add strokes to the painting but their actions do not influence the operation of the multi-robot team.

**Figure 1:**
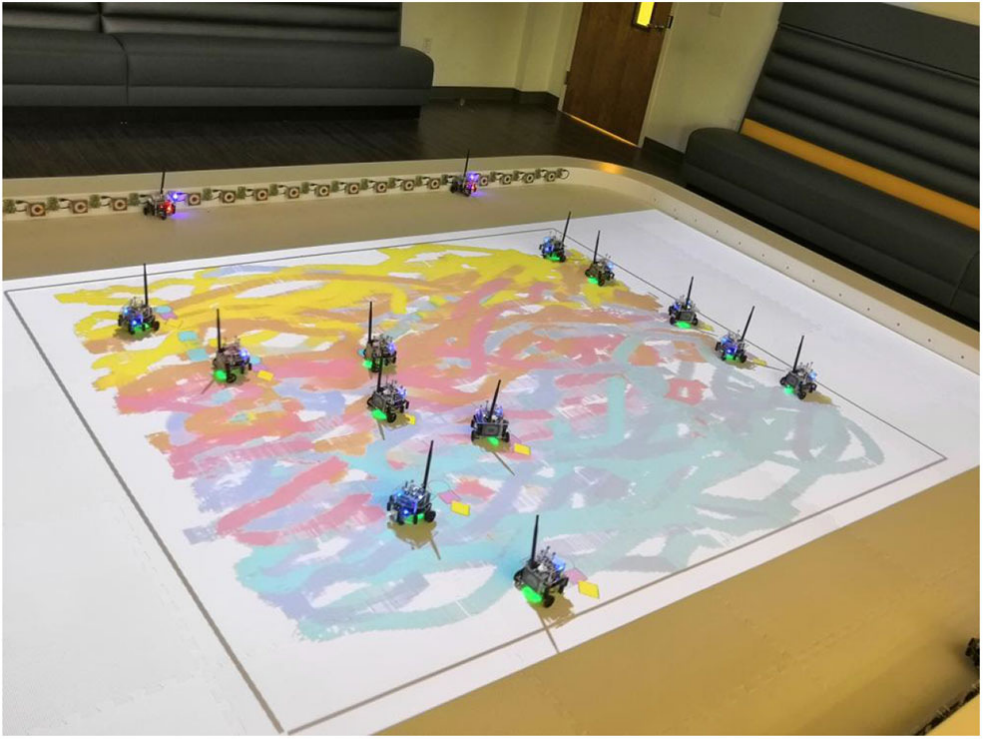
A group of 12 robots generates a painting based on the densities specified by a human user for five different colors: cyan, blue, pink, orange, and yellow. The robots lay colored trails as they move throughout the canvas, distributing themselves according to their individual painting capabilities. The painting arises as a result of the motion trails integrating over time.

In this paper, we present a multi-robot painting system based on ground robots that lay color trails as they move throughout a canvas, as shown in Figure 1. The novelty of this approach lies in the fact that a human user can influence the movement of robots capable of painting specific colors, thus controlling the concentration of certain pigments on different areas of the painting canvas. Inspired by Diaz-Mercado et al. (2015), this human-swarm interaction is formalized through the use of scalar fields—which we refer to as *density functions*—associated with the different colors such that, the higher the color density specified at a particular point, the more attracted the robots equipped with that color will be to that location. Upon the specification of the color densities, the robots move over the canvas by executing a distributed controller that optimally covers such densities taking into account the heterogeneous painting capabilities of robot team (Santos and Egerstedt, 2018; Santos et al., 2018). Thus, the system provides the human user with a high-level way to control the painting behavior of the swarm as a whole, agnostic to the total number of robots in the team or the specific painting capabilities of each of them.

The remainder of the paper is organized as follows: In section 2, we formally introduce the problem of coverage control and its extension to heterogeneous robot capabilities, as it enables the human-swarm interaction modality used in this paper. Section 3 elaborates on the generation, based on the user input, of color densities to be tracked by the multi-robot system along with the color selection strategy adopted by each robot for its colored trail. Experiments conducted on a team of differential-drive robots are presented in section 4, where different painting compositions arise as a result of various setups in terms of painting capabilities assigned to the robots. The effects of these heterogeneous resources on the final paintings are analyzed and discussed in section 5, which evaluates the color distribution in the paintings, both through color distances and chromospectroscopy, and includes a statistical analysis that illustrates the consistency of results irrespectively of initial conditions in terms of robot poses. Section 6 concludes the paper.

## 2. Density-Based Multi-Robot Control

The interactive multi-robot painting system presented in this paper operates based on the specification of desired concentration of different colors over the painting canvas. As stated in section 1, this color preeminence is encoded through color density functions that the human user can set over the domain to influence the trajectories of the robots and, thus, produce the desired coloring effect. In this section, we recall the formulation of the coverage control problem as it serves as the mathematical backbone for the human-swarm interaction modality considered in this paper.

### 2.1. Coverage Control

The coverage control problem deals with the question of how to distribute a team of *N* robots with positions $x_{i}\in {\mathbb{R}}^{d}$, i∈{1,…,N}=:N, to optimally cover the environmental features of a domain *D* ∈ ℝ^*d*^, *d* = 2 and *d* = 3 for ground and aerial robots, respectively. The question of how well the team is covering a domain is typically asked with respect to a density function, ϕ : *D* ↦ [0, ∞), that encodes the importance of the points in the domain (Cortes et al., 2004; Bullo et al., 2009). Denoting the aggregate positions of the robots as $x={\left[x_{1}^{\text{T}},\ldots ,x_{N}^{\text{T}}\right]}^{\text{T}}$, a natural way of distributing coverage responsibilities among the team is to let Robot *i* be in charge of those points closest to it,

$$\begin{array}{l} V_{i}\left(x\right)=\left\{q\in D\;|\;\|q-x_{i}\|\leq \|q-x_{j}\|,\;\forall j\in N\right\}, \end{array}$$

that is, its Voronoi cell with respect to the Euclidean distance. The quality of coverage of Robot *i* over its region of dominance can be encoded as,

(1) $$\begin{array}{l} h_{i}\left(x\right)=\int_{V_{i}\left(x\right)}{\left\|x_{i}-q\right\|}^{2}\phi \left(q\right)dq, \end{array}$$

where the square of the Euclidean distance between the position of the robot and the points within its region of dominance reflects the degradation of the sensing performance with distance. The performance of the multi-robot team with respect to ϕ can then be encoded through the locational cost in Cortes et al. (2004),

(2) $$\begin{array}{l} H\left(x\right)=\overset{N}{\underset{i=1}{\sum }}h_{i}\left(x\right)=\overset{N}{\underset{i=1}{\sum }}\int_{V_{i}\left(x\right)}{\left\|x_{i}-q\right\|}^{2}\phi \left(q\right)dq, \end{array}$$

with a lower value of the cost corresponding to a better coverage. A necessary condition for (2) to be minimized is that the position of each robot corresponds to the center of mass of its Voronoi cell (Du et al., 1999), given by

$$\begin{array}{l} C_{i}\left(x\right)=\frac{\int_{V_{i}\left(x\right)}q\phi \left(q\right)dq}{\int_{V_{i}\left(x\right)}\phi \left(q\right)dq}. \end{array}$$

This spatial configuration, referred to as a centroidal Voronoi tessellation, can be achieved by letting the multi-robot team execute the well-known Lloyd's algorithm (Lloyd, 1982), whereby

(3) $$\begin{array}{l} \dot{x_{i}}=\kappa \left(C_{i}\left(x\right)-x_{i}\right). \end{array}$$

The power of the locational cost in (2) lies on its ability to influence which areas of the domain the robots should concentrate by specifying a single density function, ϕ, irrespectively of the number of robots in the team. This makes coverage control an attractive paradigm for human-swarm interaction, as introduced in Diaz-Mercado et al. (2015), since a human operator can influence the collective behavior of an arbitrarily large swarm by specifying a single density function, e.g., drawing a shape, tapping, or dragging with the fingers on a tablet-like interface. In this paper, however, we consider a scenario where a human operator can specify multiple density functions associated with the different colors to be painted and, thus, a controller encoding such color heterogeneity must be considered. The following section recalls a formulation of the coverage problem for multi-robot teams with heterogeneous capabilities and a control law that allows the robots to optimally cover a number of different densities.

### 2.2. Coverage Control for Teams With Heterogeneous Painting Capabilities

The human-swarm interaction modality considered in this paper allows the painter to specify a set of density functions associated with different colors to produce desired concentrations of colors over the canvas. To this end, we recover the heterogeneous coverage control formulation in Santos and Egerstedt (2018). Let P be the set of paint colors and ϕ_*j*_ : *D* ↦ [0, ∞), j∈P, the family of densities associated with the colors in P defined over the convex domain, *D*, i.e., the painting canvas. In practical applications, the availability of paints given to each individual robot may be limited due to payload limitations, resource depletion, or monetary constraints. To this end, let Robot *i*, i∈N, be equipped with a subset of the paint colors, p(i)⊂P, such that it can paint any of those colors individually or a color that results from their combination. The specifics concerning the color mixing strategy executed by the robots are described in detail in section 3.

Analogously to (1), the quality of coverage performed by Robot *i* with respect to Color *j* can be encoded through the locational cost

(4) $$\begin{array}{l} h_{i}^{j}\left(x\right)=\int_{V_{i}^{j}\left(x\right)}{\left\|x_{i}-q\right\|}^{2}\phi_{j}\left(q\right)dq, \end{array}$$

where $V_{i}^{j}$ is the region of dominance of Robot *i* with respect to Color *j*. A natural choice to define the boundaries of $V_{i}^{j}$ is for Robot *i* to consider those robots in the team capable of painting Color *j* that are closest to it. If we denote as $N^{j}$ the set of robots equipped with Color *j*,

$$\begin{array}{l} N^{j}=\left\{i\in N\;|j\in p\left(i\right)\subset P\right\}, \end{array}$$

then the region of dominance of Robot *i* with respect to Color *j* ∈ *p*(*i*) is the Voronoi cell in the tessellation whose generators are the robots in $N^{j}$,

$$\begin{array}{l} V_{i}^{j}\left(x\right)=\left\{q\in D\;|\;\|x_{i}-q\|\leq \|x_{k}-q\|,\forall k\in N^{j}\right\}. \end{array}$$

**Figure 2:**
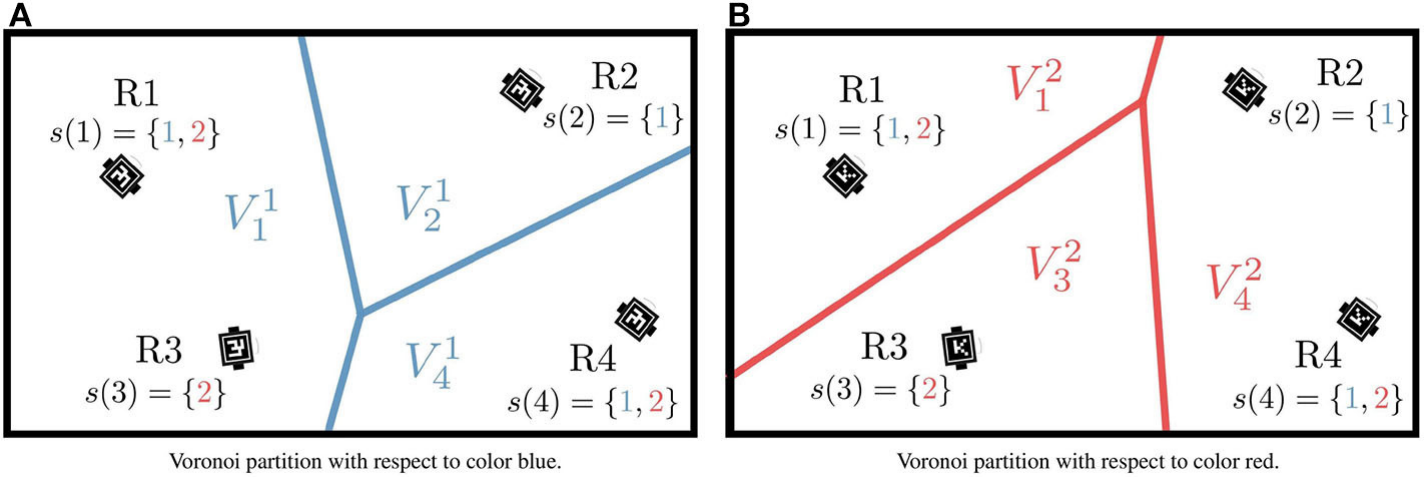
Regions of dominance for four neighboring robots with respect to colors blue (1) **(A)**, and red (2) **(B)**. For each color, the resulting Voronoi cells are generated only by those robots equipped with that painting color. Source: Adapted from Santos and Egerstedt (2018).

Note that, if Robot *i* is the only robot equipped with Color *j*, then the robot is in charge of covering the whole canvas, i.e., $V_{i}^{j}=D$. Under this partition strategy, as illustrated in Figure 2, the area that Robot *i* is responsible for with respect to Color *j*, $V_{i}^{j}$, can differ from the region to be monitored with respect to Color *k*, $V_{i}^{k}$, *j, k* ∈ *p*(*i*).

With the regions of dominance defined, we can now evaluate the cost in (4). Thus, the overall performance of the team can be evaluated by considering the complete set of robots and color equipments through the *heterogeneous locational cost* formulated in Santos and Egerstedt (2018),

(5) $$\begin{array}{l} H_{het}\left(x\right)=\underset{j\in P}{\sum }\underset{i\in N^{j}}{\sum }\int_{V_{i}^{j}\left(x\right)}{\left\|x_{i}-q\right\|}^{2}\phi_{j}\left(q\right)dq, \end{array}$$

with a lower value of the cost corresponding to a better coverage of the domain with respect to the family of color density functions ϕ_*j*_, j∈P.

Letting Robot *i* follow a negative gradient descent of $H_{het}$ establishes the following control law.

**Theorem** 1 (Heterogeneous Gradient Descent, Santos and Egerstedt, 2018). *Let Robot *i*, with planar position *x*_*i*_, evolve according to the control law* $\dot{x_{i}}=u_{i}$, *where*

(6) $$\begin{array}{l} u_{i}=\kappa \underset{j\in p\left(i\right)}{\sum }M_{i}^{j}\left(x\right)\left(C_{i}^{j}\left(x\right)-x_{i}\right), \end{array}$$

*with* $M_{i}^{j}\left(x\right)$ *and* $C_{i}^{j}\left(x\right)$, *respectively, the *heterogeneous mass* and *center of mass* of Robot *i* with respect to Color *j*, defined as*

(7) $$\begin{array}{l} M_{i}^{j}\left(x\right)=\int_{V_{i}^{j}\left(x\right)}\phi_{j}\left(q\right)dq,\;C_{i}^{j}\left(x\right)=\frac{\int_{V_{i}^{j}\left(x\right)}q\phi_{j}\left(q\right)dq}{M_{i}^{j}\left(x\right)}. \end{array}$$

*Then, as t* → ∞, *the robots will converge to a critical point of the heterogeneous locational cost in (5) under a positive gain κ > 0*.

*Proof*: See Santos and Egerstedt (2018).

Therefore, the controller that minimizes the heterogeneous locational cost in (5) makes each robot move according to a weighted sum where each term corresponds with a continuous-time Lloyd descent—analogous to (3)—over a particular color density ϕ_*j*_, weighted by the mass corresponding to that painting capability.

The controller in (6) thus enables an effective human-swarm interaction modality for painting purposes where the human painter only has to specify color density functions for the desired color composition and the controller allows the robots in the team to distribute themselves over the canvas according to their heterogeneous painting capabilities. Note that, while other human-swarm interaction paradigms based on coverage control have considered time-varying densities to model the input provided by an external operator (Diaz-Mercado et al., 2015), in the application considered in this paper heterogeneous formulation of the coverage control problem, while considering static densities, suffices to model the information exchange between the human and the multi-robot system.

## 3. From Coverage Control to Painting

In section 2, we established a human-swarm interaction paradigm that allows the user to influence the team of robots so that they distribute themselves throughout the canvas according to a desired distribution of color and their painting capabilities. But how is the painting actually created? In this section, we present a strategy that allows each robot to choose the proportion in which the colors available in its equipment should be mixed in order to produce paintings that reflect, to the extent possible, the distributions of color specified by the user.

The multi-robot system considered in this paper is conceived to create a painting by means of each robot leaving a trail of color as it moves over a white canvas. While the paintings presented in section 4 do not use physical paint but, rather, projected trails over the robot testbed, the objective of this section is to present a color model that both allows the robots to produce a wide range of colors with minimal painting equipment and that closely reflects how the color mixing would occur in a scenario where physical paint were to be employed. To this end, in order to represent a realistic scenario where robots lay physical paint over a canvas, we use the subtractive color mixing model (see Berns, 2000 for an extensive discussion in color mixing), which describes how dyes and inks are to be combined over a white background to absorb different wavelengths of white light to create different colors. In this model, the primary colors that act as a basis to generate all the other color combinations are cyan, magenta, and yellow (CMY).

The advantage of using a simple model like CMY is two-fold. Firstly, one can specify the desired presence of an arbitrary color in the canvas by defining in which proportion these should mix at each point and, secondly, the multi-robot system as a collective can generate a wide variety of colors being equipped with just cyan, magenta and yellow paint, i.e., P={C,M,Y} in the heterogeneous multi-robot control strategy in section 2.2. The first aspect reduces the interaction complexity between the human and the multi-robot system: the painter can specify a desired set of colors C throughout the canvas by defining the CMY representation of each color β∈C as $\left[\beta_{C},\beta_{M},\beta_{Y}\right],\beta_{j}\in \left[0,1\right],j\in P$, and its density function over the canvas ϕ_β_(*q*), *q* ∈ *D*. Note that a color specified in the RGB color model (red, green, and blue), represented by the triple [β_*R*_, β_*G*_, β_*B*_], can be directly converted to the CMY representation by subtracting the RGB values from 1, i.e., [β_*C*_, β_*M*_, β_*Y*_] = 1 − [β_*R*_, β_*G*_, β_*B*_]. Given that the painting capabilities of the multi-robot system are given by P={C,M,Y}, the densities that the robots are to cover according to the heterogeneous coverage formulation in section 2.2 can be obtained as,

$$\begin{array}{l} \phi_{j}\left(q\right)=\underset{\beta \in C}{⊕}\beta_{j}\phi_{\beta }\left(q\right),\;j\in P, \end{array}$$

where ⊕ is an appropriately chosen composition operator. The choice of composition operator reflects how the densities associated with the different colors should be combined in order to compute the overall density function associated with each CMY primary color. For example, one way to combine the density functions is to compute the maximum value at each point,

$$\begin{array}{l} \phi_{j}\left(q\right)=\underset{\beta \in C}{\max}\beta_{j}\phi_{\beta }\left(q\right),\;j\in P. \end{array}$$

The question remaining is how a robot should combine its available pigments in its color trail to reflect the desired color density functions. The formulation of the heterogeneous locational cost in (5) implies that Robot *i* is in charge of covering Color *j* within the region dominance $V_{i}^{j}$ and of covering Color *k* within $V_{i}^{k}$, j,k∈p(i)⊂P. However, depending on the values of the densities ϕ_*j*_ and ϕ_*k*_ within these Voronoi cells, the ratio between the corresponding coverage responsibilities may be unbalanced. In fact, such responsibilities are reflected naturally through the heterogeneous mass, $M_{i}^{j}\left(x\right)$, defined in (7). Let us denote as $[\alpha_{i}^{C}$, $\alpha_{i}^{M}$, $\alpha_{i}^{Y}]$, $\alpha_{i}^{j}\in \left[0,1\right]$, $\alpha_{i}^{C}+\alpha_{i}^{M}+\alpha_{i}^{Y}=1$, the color proportion in the CMY basis to be used by Robot *i* in its paint trail. Then, a color mixing strategy that reflects the coverage responsibilities of Robot *i* can be given by,

(8) $$\begin{array}{l} \alpha_{i}^{j}=\frac{M_{i}^{j}\left(x\right)}{\sum_{k\in p\left(i\right)}M_{i}^{k}\left(x\right)},\;j\in p\left(i\right)\subset P. \end{array}$$

Note that, when $M_{i}^{j}\left(x\right)=0,\forall j\in p\left(i\right)\subset P$, the robot is not covering any density and, thus, $\alpha_{i}^{j},j\in P$, can be undefined.

**Figure 3:**
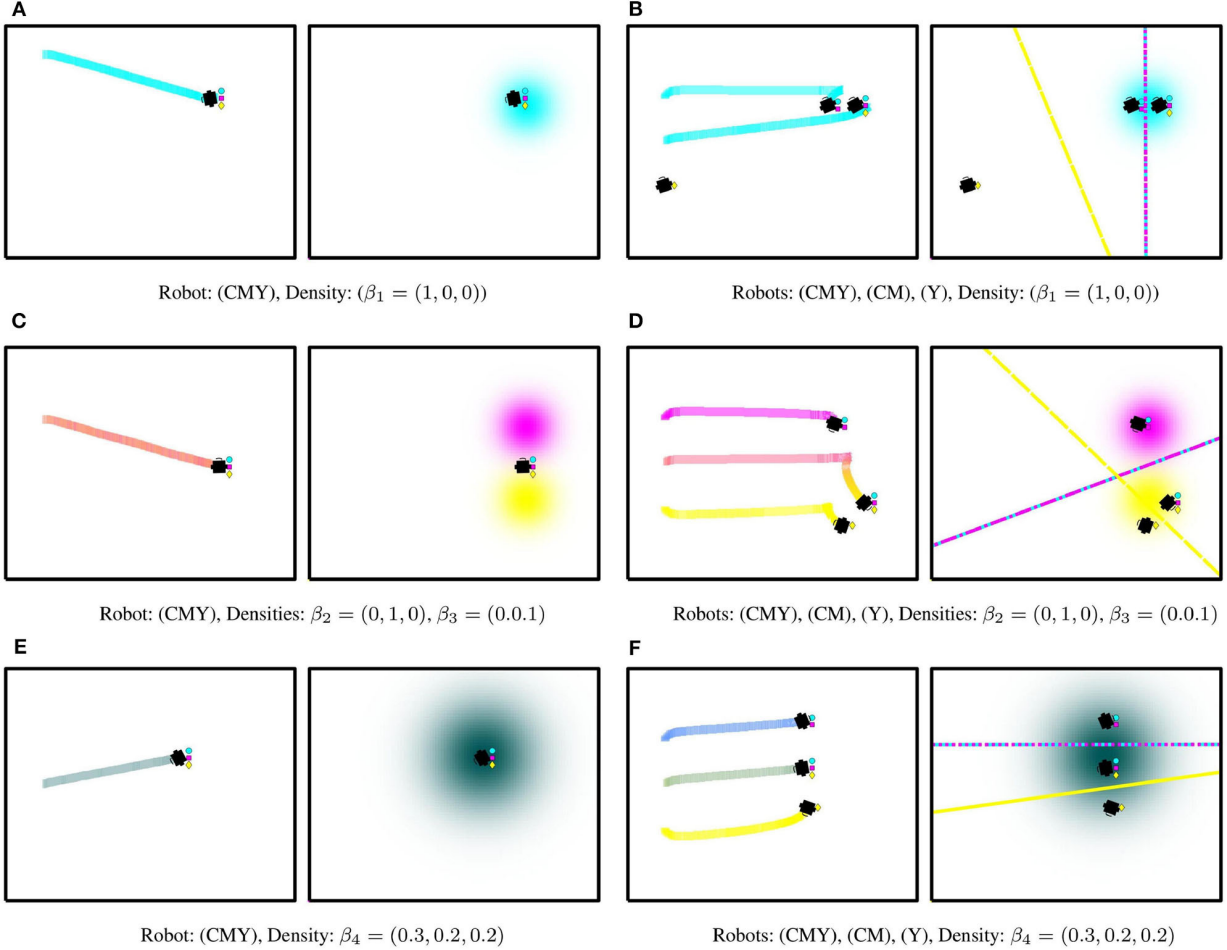
Painting mechanism based on heterogeneous coverage control. Each subfigure shows the color trails laid by the robots (left) as they move to optimally cover a user-specified color density function (right) by executing the controller in (6). The symbols located to the right of the robot indicate its painting capabilities. **(A,C,E)** Show the operation of the painting mechanism in section 3 for a single robot equipped with all three colors, i.e., cyan (C), magenta (M), and yellow (Y), thus capable of producing all color combinations in the CMY basis. In **(A)**, the robot lays a cyan trail according to the density color specification β_1_. The robot equally mixes magenta and yellow in **(C)** according to the color mixing strategy in (8), producing a color in between the two density color specifications, β_2_ and β_3_. Finally, in **(E)**, the robot exactly replicates the color specified by β_4_. On the other hand, **(B,D,F)** depict the operation of the painting mechanism with a team of 3 robots, where the Voronoi cells (color coded according to the CMY basis) are shown on the density subfigures.

Figure 3 illustrates the operation of this painting mechanism for three different density color specifications. Firstly, the mechanism is simulated for a robot equipped with all three colors—cyan (C), magenta (M), and yellow (Y)—in Figures 3A,C,E. As seen, the robot lays a cyan trail as it moves to optimally cover a single cyan density function in Figure 3A. In Figure 3C, two different density functions are specified, one magenta and one yellow, and the robot lays down a trail whose color is a combination of both paints. Finally, in Figure 3E, the robot is tasked to cover a density that is a combination of the CMY colors. Since the robot is equipped with all three colors, the trail on the canvas exactly replicates the colors desired by the user.

For the same input density specifications, Figures 3B,D,F illustrate the trails generated by a team of three robots equipped with different subsets of the color capabilities. As seen, the color of the individual robot trails evolve as a function of the robot's equipment, the equipments of its neighbors, and the specified input density functions. A simulation depicting the operation of this painting mechanism can be found in the video included in the Supplementary Materials.

## 4. Experimental Results With Projected Trails

The proposed multi-robot painting system is implemented on the Robotarium, a remotely accessible swarm robotics testbed at the Georgia Institute of Technology (Wilson et al., 2020). The experiments, uploaded via web, are remotely executed on a team of up to 20 custom-made differential-drive robots. On each iteration, run at a maximum rate of 120 Hz, the Robotarium provides the poses of the robots, tracked by a motion capture system, and allows the control program to specify the linear and angular velocities to be executed by each robot in the team. An overhead projector affords the visualization of time-varying images onto the test bed during the execution of the experiments. The data is made available to the user once the experiment is finalized.

The human-swarm interaction paradigm for color density coverage presented in section 2 and the trail color mixing strategy from section 3 are illustrated experimentally on a team of 12 robots over a 2.4 × 2m canvas. The robots lay trails of color as they cover a set of user-defined color density functions according to the control law in (6), where κ = 1 for all the experiments and the single integrator dynamics $u_{i},i\in N$ are converted into linear and angular velocities executable by the robots using the near-identity diffeomorphism from Olfati-Saber (2002), a functionality available in the Robotarium libraries. In order to study how the limited availability of painting resources affects the resulting painting, for the same painting task, nine different experimental setups in terms of paint equipment assigned to the multi-robot team are considered. While no physical paint is used in the experiments included in this paper, the effectiveness of the proposed painting system is illustrated by visualizing the robots' motion trails over the canvas with an overhead projector.

The experiment considers a scenario where the multi-robot team has to simultaneously cover a total of six different color density functions over a time horizon of 300 s. These density functions aim to represent commands that would be interactively generated by the user, who would be observing the painting being generated and could modify the commands for the color densities according to his or her artistic intentions. Note that, in this paper, these time-varying density functions are common to all the experiments and simulations included in sections 4, 5 to allow the evaluation of the paintings as a function of the equipment setups in **Table 2**. In an interactive scenario, the density commands are to be generated in real time by the user, by means of a tablet-like interface, for example. In this experiment, the color density functions involved are of the form,

(9) $$\begin{array}{l} \phi_{\beta }\left(q\right)=\frac{K}{2\pi \sigma_{x}\sigma_{y}}\exp\left(-\frac{{\left(q_{x}-{\bar{\mu }}_{x}\right)}^{2}+{\left(q_{y}-{\bar{\mu }}_{y}\right)}^{2}}{2\sigma_{x}^{2}\sigma_{y}^{2}}\right), \end{array}$$

with β∈{1,…,6}=C, *q* = [*q*_*x*_, *q*_*y*_]^T^ ∈ *D*. The color associated with each density as well as its parameters are specified in Table 1, and ${\bar{\mu }}_{x}$ and ${\bar{\mu }}_{y}$ are given by

$$\begin{array}{l} {\bar{\mu }}_{x}=\mu_{x}-A_{x}\sin\left(2\pi f_{x}t\right),\\ {\bar{\mu }}_{y}=\mu_{y}-A_{y}\sin\left(2\pi f_{y}t\right). \end{array}$$

**Figure 4:**
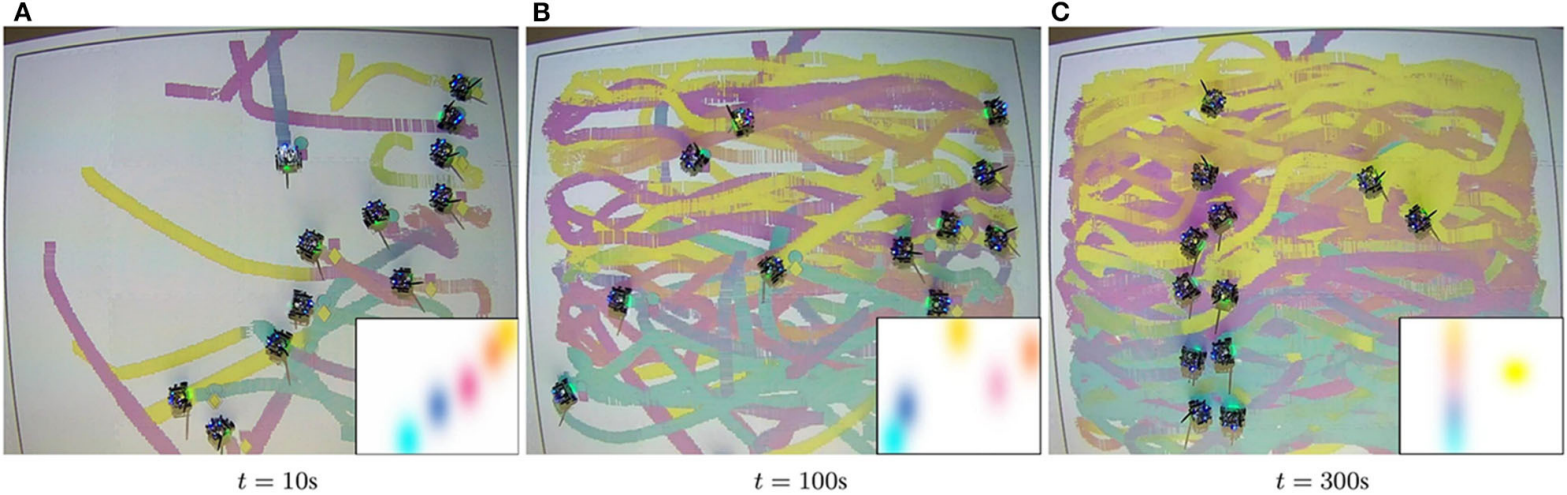
Evolution of the painting according to the density parameters in Table 1, for the Setup 3 given as in Table 2. The robots distribute themselves over the domain in order to track the density functions as they evolve through the canvas. In each snapshot, the densities that the multi-robot team is tracking at that specific point in time are depicted in the bottom right corner of the image. The color distribution of the color trails reflects the colors specified for the density functions within the painting capabilities of the robots. Even though none of the robots is equipped with the complete CMY equipment and, thus, cannot reproduce exactly the colors specified by the user, the integration of the colors over time produce a result that is close to the user's density specification.

Figure 4 illustrates the evolution of the painting for a specific equipment setup as the robots move to cover these densities at *t* = 100s and *t* = 300s.

**Table 1:** Experimental parameters associated with the user-specified color density functions.

**β**	**Color**	**β_C_**	**β_M_**	**β_Y_**	**K**	**μ_x_**	**μ_y_**	**σ_x_**	**σ_y_**	***A*_x_**	***A*_y_**	***f*_x_**	***f*_y_**
1	Yellow	0.0000	0.0863	0.5569	60	0	0.8	0.22	0.22	1.1	0.1	1/40	0
2	Orange	0.0000	0.3529	0.5569	40	0	0.4	0.22	0.22	1.1	0.1	1/37	2/15
3	Pink	0.0549	0.5529	0.3451	40	0	0	0.22	0.22	1.1	0.1	1/35	0
4	Blue	0.4314	0.3098	0.1373	60	0	−0.4	0.22	0.22	1.1	0.1	1/33	2/15
5	Cyan	0.9686	0.0353	0.0275	40	0	−0.8	0.22	0.22	1.1	0.1	1/30	0
6	Yellow Sun	0	0	1	60	0.5	0.3	0.125	0.125	0.1	0.1	1/5	1/5

**Figure 5:**
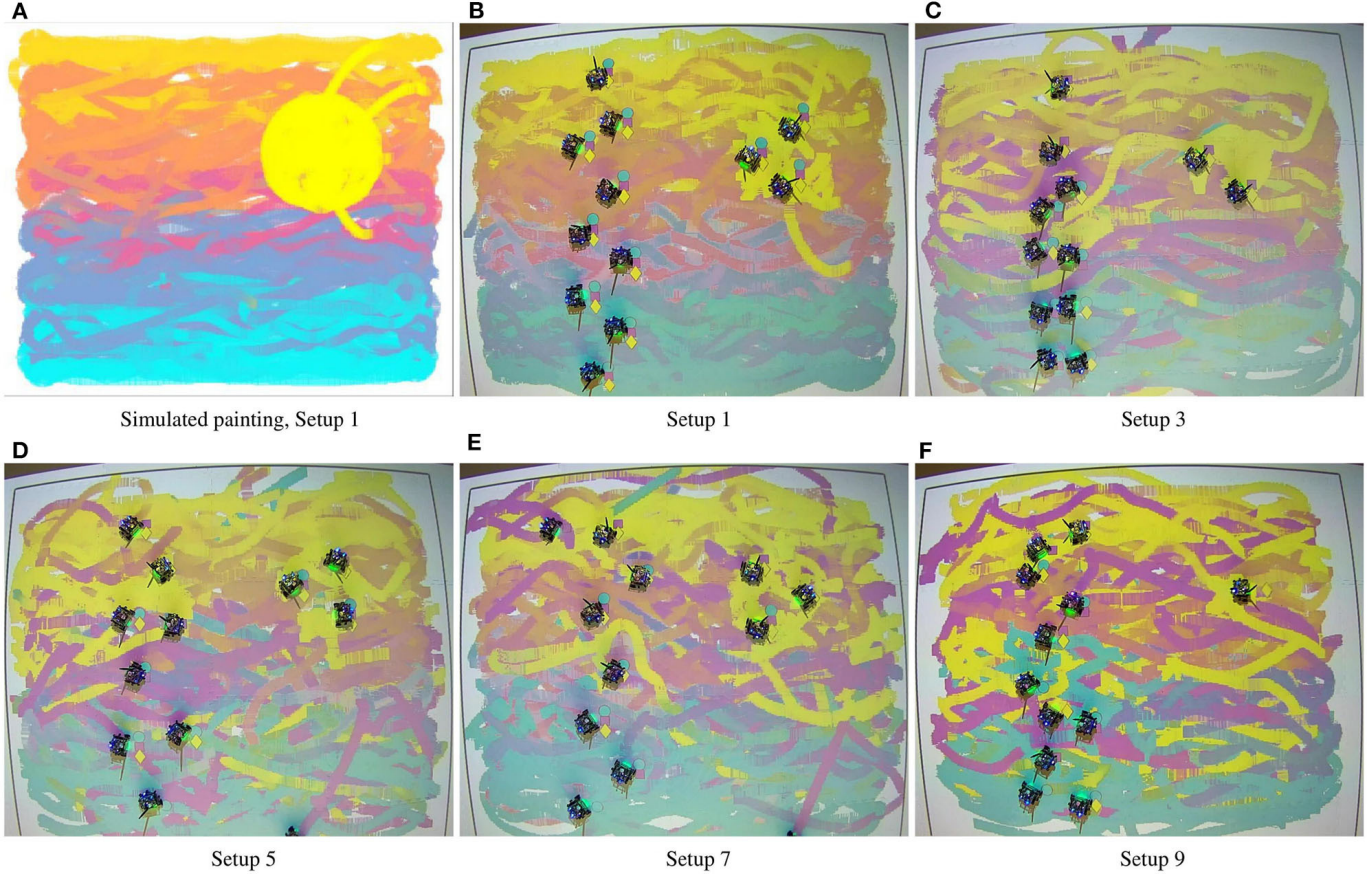
Paintings generated for the densities in (9), with the team of 12 robots in their final positions. **(A)** Corresponds to a simulated painting and it is used for benchmarking. According to the painting equipment setups in Table 2 we can see how, as the robots in the team are equipped with more painting capabilities, the color gradients become smoother and more similar to the ideal outcome.

The multi-robot painting strategy is evaluated under a series of painting equipment setups to assess the differences that result from the heterogeneity of the team, which can be motivated by the scarcity or depletion of painting resources or by a design choice of the human user, for example. Table 2 outlines the color painting capabilities available to each of the robots in the different experimental setups. The paintings which result from five of these configurations (the ones with an odd setup ID) are shown in Figure 5. The generative process for the paintings in Figure 5 is illustrated in the video included in the Supplementary Materials. Note that all these experiments where run with identical initial conditions in terms of robot poses, according to the identifiers in Table 2. For the purpose of benchmarking, a simulated painting is generated for painting setup 1, i.e., with a homogeneous equipment capable of reproducing any color. This simulated painting is created under the same heterogeneous density coverage control and color mixing strategies as in the robotic experiments, but considering unicycle dynamics without actuator limits or saturation and with no communication delays (Figure 5A). Given the paintings in Figures 5B–F, we can observe how the closest color distribution to the simulated painting is achieved in Figure 5B, which corresponds to the case where all the robots have all the painting capabilities—i.e., the team is homogeneous—and, thus, can reproduce any combination of colors in the CMY basis.

**Table 2:** Paint equipment for the different experimental setups.

**Setup**	**Paint equipment**																	**Heterogeneity**	
**ID**	**ID**	**1**	**2**	**3**	**4**	**5**	**6**	**7**	**8**	**9**	**10**	**11**	**12**	**13**	**14**	**15**	**Total**	**Sunset**	**8-bit RGB**
1	C	×	×	×	×	×	×	×	×	×	×	×	×				12	0	0
	M	×	×	×	×	×	×	×	×	×	×	×	×				12	
	Y	×	×	×	×	×	×	×	×	×	×	×	×				12	
2	C	×			×		×	×	×	×	×	×	×	×	×	×	12	0.2786	0.2680
	M		×		×	×		×	×	×	×	×	×	×	×	×	12	
	Y			×		×	×	×	×	×	×	×	×	×	×	×	12	
3	C	×	×			×	×	×	×			×	×				8	0.3060	0.2963
	M	×	×	×	×			×	×	×	×						8
	Y			×	×	×	×			×	×	×	×				8
4	C	×		×	×	×		×		×	×	×	×				9	0.3340	0.3121
	M		×	×		×	×	×	×		×	×	×				9
	C		×	×		×	×		×	×	×	×	×				9
5	C	×			×		×	×	×	×	×	×	×				9	0.3921	0.3783
	M		×		×	×		×	×	×	×	×	×				9
	Y			×		×	×	×	×	×	×	×	×				9
6	C	×	×					×	×	×	×	×	×				8	0.4488	0.4398
	M			×	×			×	×	×	×	×	×				8
	Y					×	×	×	×	×	×	×	×				8
7	C	×			×	×			×	×	×	×	×				8	0.5686	0.5498
	M		×		×	×	×	×			×	×	×				8
	Y			×			×	×	×	×	×	×	×				8
8	C	×	×	×							×	×	×				6	0.6904	0.6835
	M				×	×	×				×	×	×				6
	Y							×	×	×	×	×	×				6
9	C	×	×					×	×			×	×				6	0.8148	0.8004
	M			×	×			×	×	×	×						6
	Y					×	×			×	×	×	×				6

It is interesting to note the significant changes in the characteristics of the painting for different equipment configurations of the robots. For equipment setups 3, 5, 7, and 9, where some robots—or all—are not equipped with all the color paints, the corresponding paintings do not show as smooth color gradients as the one in Figure 5B. However, the distribution of color for these paint setups still qualitatively reflects the color specification given by the densities in Table 1. Even in the extreme case of Equipment 9 (see Figure 5F), where none of the robots is equipped with all CMY paints—in fact, half of the robots only have one paint and the other half have pairwise combinations—the robot team still renders a painting that, while presenting colors with less smooth blending than the other setups, still represents the color distribution specified by the densities in Table 1. For Setups 3 and 7, the team has the same total number of CMY painting capabilities but the distribution is different among the team members: in Setup 3 none of the robots are equipped with the three colors, while in Setup 7 there are some individuals that can paint any CMY combination and others can paint only one color. Observing the Figures 5C,E, while the resulting colors are less vibrant for the equipment in Setup 3, there seems to be a smoother blending between them along with the vertical axis. Setup 7 produces a painting where overall the colors are more faithful to the ideal outcome presented in Figure 5A, but that also contain stronger trails corresponding to the pure primary colors appear throughout the painting. If we compare Figures 5D,E we can see how, by adding a small amount of painting capabilities to the system, the color gradients are progressively smoothed. This observation suggests to further analyze the variations that appear on the paintings as a function of the heterogeneous equipment configurations of the different setups. This will be the focus of the next section.

## 5. Analysis and Discussion

As described in section 1, the robotic painting system developed in this paper generates illustrations via an interaction between the color density functions specified by the user and the different color equipment present on the robots. In particular, the different equipments not only affect the color trails left by the robots, but also affect their motion as they track the density functions corresponding to their equipment. While Figure 5 qualitatively demonstrates how the nature of the painting varies with different equipment setups, this section presents a quantitative analysis of the variations among paintings resulting from different equipment setups. We also analyze the reproducibility characteristics of the multi-robot painting system, by investigating how paintings vary among different realizations using the same equipment setups.

Let *S* denote the number of distinct equipment setups of the robots in the team—where each unique configuration denotes a robot *species*. We denote *s*_ι_ ∈ [0, 1] as the probability that a randomly chosen agent belongs to species ι, ι∈S={1,…,S}, such that

$$\begin{array}{l} \overset{S}{\underset{\iota =1}{\sum }}s_{\iota }=1,\;\text{and }s={\left[s_{1},\ldots ,s_{S}\right]}^{\text{T}}. \end{array}$$

For each equipment setup in Table 2, these probabilities can be calculated as a function of how many agents are equipped with each subset of the paint colors.

We adopt the characterization developed in Twu et al. (2014), and quantify the heterogeneity of a multi-robot team as,

(10) $$\begin{array}{l} H\left(s\right)=E\left(s\right)Q\left(s\right), \end{array}$$

where *E*(*s*) represents the *complexity* and *Q*(*s*), the *disparity* within the multi-robot system for a given experimental setup, *s*. More specifically, *E*(*s*) can be modeled as the *entropy* of the multi-agent system,

$$\begin{array}{l} E\left(s\right)=-\overset{S}{\underset{\iota =1}{\sum }}s_{\iota }\log\left(s_{\iota }\right), \end{array}$$

and *Q*(*s*) is the *Rao's Quadratic Entropy*,

(11) $$\begin{array}{l} Q\left(s\right)=\overset{S}{\underset{\iota =1}{\sum }}\overset{S}{\underset{\kappa =1}{\sum }}s_{\iota }s_{\kappa }\delta {\left(\iota ,\kappa \right)}^{2}, \end{array}$$

with $\delta :S\times S\mapsto {\mathbb{R}}_{+}$ a metric distance between species of robots. More specifically, δ represents the differences between the abilities of various species *in the context of performing a particular task*. For example, if we have three robots, one belonging to species *s*_5_ (*p*(*s*_5_) = {*C*}) and two belonging to species *s*_8_ (*p*(*s*_8_) = {*C, M, Y*}) and we have to paint only cyan, then the distance between agents should be zero, since all of them can perform the same task. However, if the task were to paint a combination of yellow and magenta, then the species *s*_5_ could not contribute to that task and, therefore, δ > 0.

Similar to Twu et al. (2014), we formalize this idea by introducing a task space, represented by the tuple (*T*, γ) where *T* denotes the set of tasks, and γ:*T*↦ℝ_+_ represents an associated weight function. In this paper, the set of tasks *T* simply correspond to the different colors specified by the user, as shown in Table 1. Consequently, a task $t_{\beta }^{j}\in T$ corresponds to the component *j*, *j* ∈ {*C, M, Y*}, of color input β∈C. The corresponding weight functions for the tasks are calculated as,

$$\begin{array}{l} \gamma \left(t_{\beta }^{j}\right)=\frac{\beta_{j}}{\underset{\tilde{\beta }\in C}{\sum }\underset{k\in P}{\sum }{\tilde{\beta }}_{k}}. \end{array}$$

With this task-space, the task-map, $\omega :S\mapsto 2^{T}$, as defined in Twu et al. (2014), directly relates the different robot species with the CMY colors, i.e., if the color equipment of species ι is denoted as *p*(ι), then it can execute tasks $t_{\beta }^{j}$ if *j* ∈ *p*(ι).

Having defined the task-space, (*T*, γ), and the task-map, ω, the distance between two agents *i* and *j* can be calculated as in Twu et al. (2014),

$$\begin{array}{l} \delta \left(T,\gamma ,\omega \right)\left(\iota ,\kappa \right)=\frac{\underset{t\in \left(\omega \left(\iota \right)\cup \omega \left(\kappa \right)\right)\backslash \left(\omega \left(\iota \right)\cap \omega \left(\kappa \right)\right)}{\sum }\gamma \left(t\right)}{\underset{u\in \left(\omega \left(\iota \right)\cup \omega \left(\kappa \right)\right)}{\sum }\gamma \left(u\right)}. \end{array}$$

This task-dependent distance metric between different robot species can then be used to compute the *disparity* as shown in (11).

Having completely characterized the disparity, *Q*(*s*), and the complexity, *E*(*s*), of an experimental setup under a specific painting task, one can compute the heterogeneity measure associated with them according to (10). To this end, the third column in Table 2 represents the heterogeneity measure of the different setups. The heterogeneity values have been computed for the sunset-painting task from Table 1, as well as for a generic painting task that considers the whole 8-bit RGB color spectrum as objective colors to be painted by the team. This latter task is introduced in this analysis with the purpose of serving as a baseline to evaluate the comprehensiveness of the proposed sunset painting task. As it can be observed in Table 2, the heterogeneity values obtained for the sunset and the 8-bit RGB tasks are quite similar and the relative ordering of the setups with respect to the heterogeneity measure is the same, thus suggesting that the sunset task used in this paper requires a diverse enough set of painting objectives for all the equipment setups proposed. Armed with this quantification of team heterogeneity, we now analyze how the spatial characteristics of the painting differ as the equipment configurations change.

### 5.1. Color Distance

We first analyze the complex interplay between motion trails and equipment setups by computing the spatial distance between the mean location of the desired input density function specified by the user, and the resulting manifestation of the color in the painting. To this end, we use the *color distance* metric introduced in Androutsos et al. (1998) to characterize the distance from the color obtained in every pixel of the resulting painting to each of the input colors specified in Table 1.

Let ρ(*q*) represent the 8-bit RGB vector value for a given pixel *q* in the painting. Then, the color distance between two pixels *q*_*i*_ and *q*_*j*_ is given as,

(12) $$\begin{array}{l} d_{p}\left(q_{i},q_{j}\right)=1-\left[1-\frac{2}{\pi }\cos^{-1}\left(\frac{\rho \left(q_{i}\right)\cdot \rho \left(q_{j}\right)}{\left\|\rho \left(q_{i}\right)\|\|\rho \left(q_{j}\right)\right\|}\right)\right]\\ \;\left[1-\frac{\left\|\rho \left(q_{i}\right)-\rho \left(q_{j}\right)\right\|}{\sqrt{3\cdot 255^{2}}}\right] \end{array}$$

Using (12), we can compute the distance from the color of each pixel to each of the input colors specified by the user (given in this paper by Table 1). For a given pixel in the painting *q* and input color β, these distances can be interpreted as a color-distance density function over the domain, denoted as φ

$$\begin{array}{l} \varphi \left(q,\beta \right)=\exp\left(-\frac{d_{p}\left(q,\beta \right)}{\varsigma^{2}}\right), \end{array}$$

where, with an abuse of notation, *d*_*p*_(*q*, β) represents the color distance between the color β and the color at pixel *q*. For the experiments conducted in this paper, ς^2^ was chosen to be 0.1.

Since we are interested in understanding the spatial characteristics of colors in the painting, we compute the center of mass of a particular color β in the painting,

(13) $$\begin{array}{l} C_{\beta }=\frac{\int_{D}q\varphi \left(q,\beta \right)dq}{\int_{D}\varphi \left(q,\beta \right)dq}. \end{array}$$

The covariance ellipse for the color β at a pixel *q* is given as,

(14) $$\begin{array}{l} V_{\beta }\left(q\right)=\sqrt{\left(\varphi \left(q\right)\right)}\left(q-C_{\beta }\right). \end{array}$$

**Figure 6:**
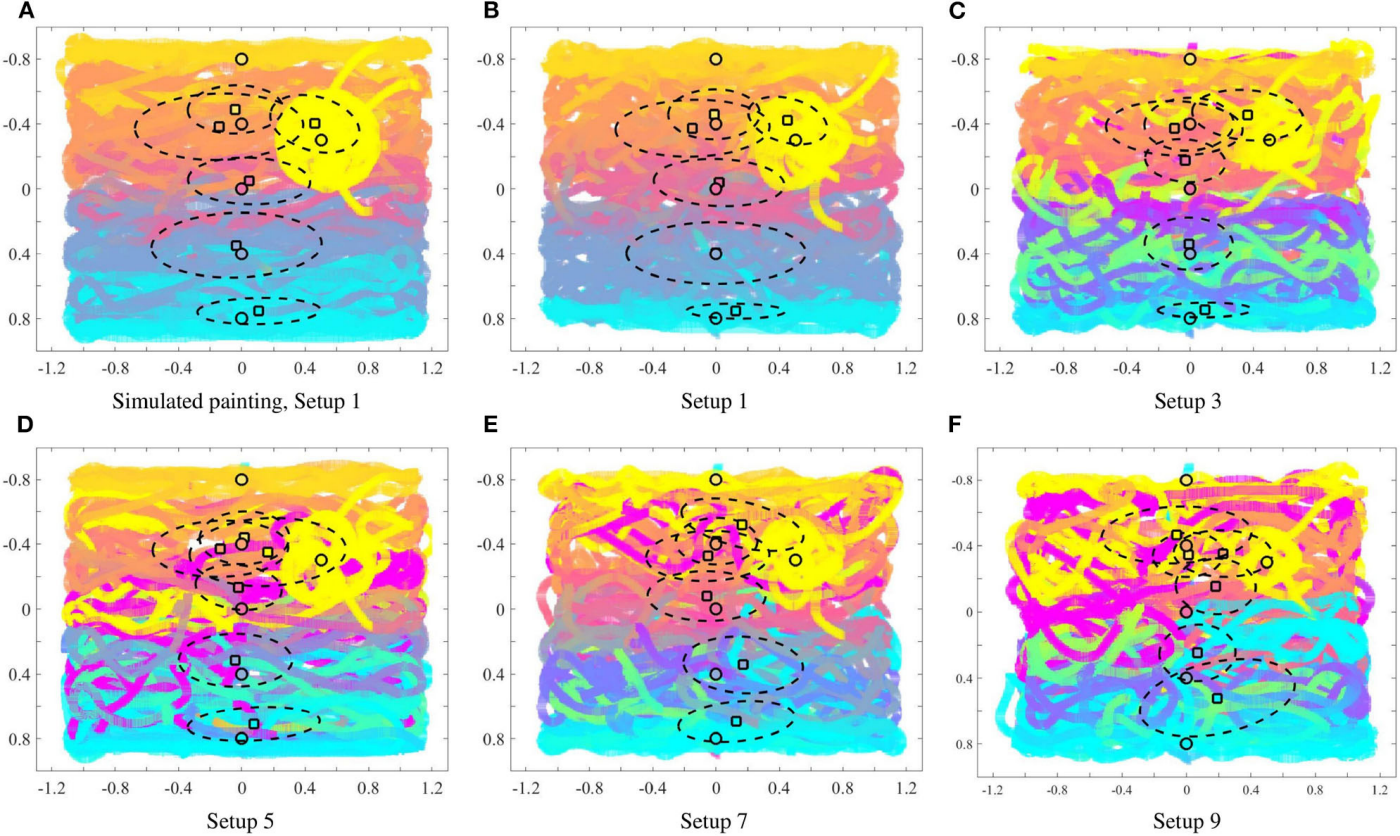
For each input color (given in Table 1): mean of the input density function (circle), and center of mass of the resulting color according to (13) (square). The dotted lines depict the covariance ellipse according to (14). As seen the heterogeneity of the multi-robot team [as defined in (10)] impacts how far the colors are painted from the location of the input, as given by the user.

**Figure 7:**
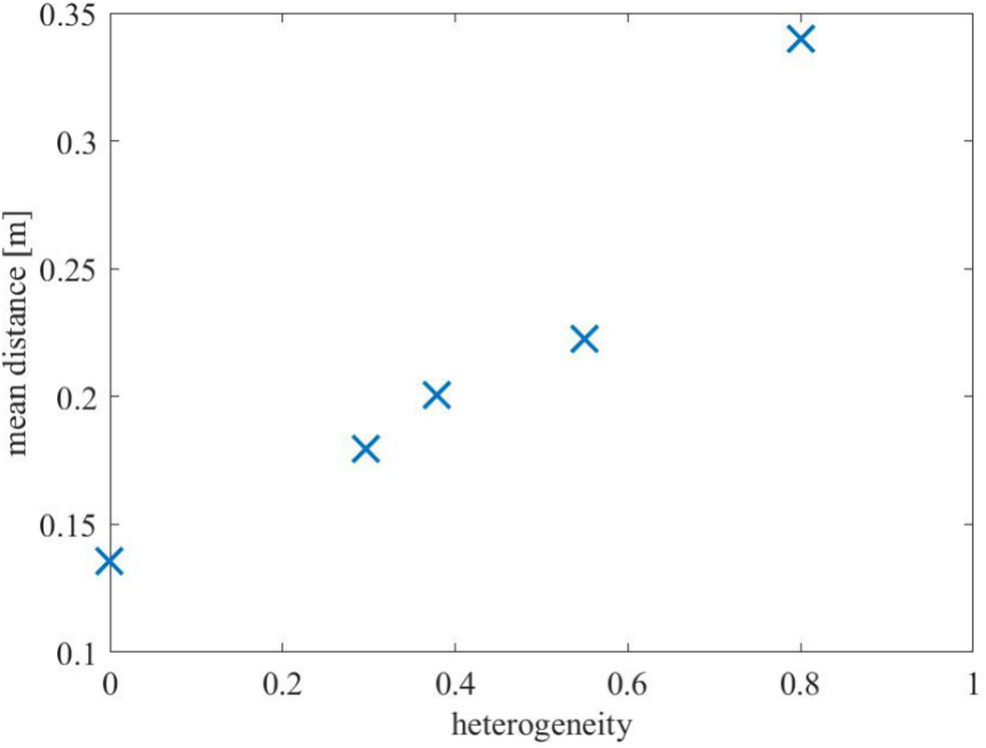
Average distance from mean density input to the resulting center of mass over the input colors of the painting as a function of the heterogeneity among the robots [as defined in (10)]. As seen, with increasing sparsity of painting equipment on the robots (signified by increasing heterogeneity), the mean distance increases, indicating that colors get manifested farther away from where the user specifies them.

For each of the input colors, Figure 6 illustrates the extent to which the color center of masses (computed by (13) and depicted by the square filled by the corresponding color) are different from the mean locations of the input density functions (depicted by the circle). For all the painting equipment setups in Figure 6, as the heterogeneity of the team increases, the mean of the input density function for each color and the resulting center of mass become progressively more distant. This phenomenon is illustrated in Figure 7, where the mean distance between the input density and the resulting color center of mass is plotted as a function of the heterogeneity of the equipment of the robots. For a given painting *P*, this distance is computed as,

(15) $$\begin{array}{l} d_{c}\left(P\right)=\frac{\sum_{\beta \in C}\|\mu_{\beta }-C_{\beta }\|}{\left|C\right|}, \end{array}$$

where C represents the set of input colors, and μ_β_ represents the mean of the input density function for color β. As seen, with increasing heterogeneity, the mean distance increases because lesser painting capabilities on the robots do not allow them to exactly reproduce the input color distributions. However, even with highly heterogeneous setups, such as Setups 7 or 9, the multi-robot team is still able to preserve highly distinguishable color distributions throughout the canvas, which suggests that the coverage control paradigm for multi-robot painting is quite robust to highly heterogeneous robot teams and resource deprivation.

### 5.2. Chromospectroscopy

**Figure 8:**
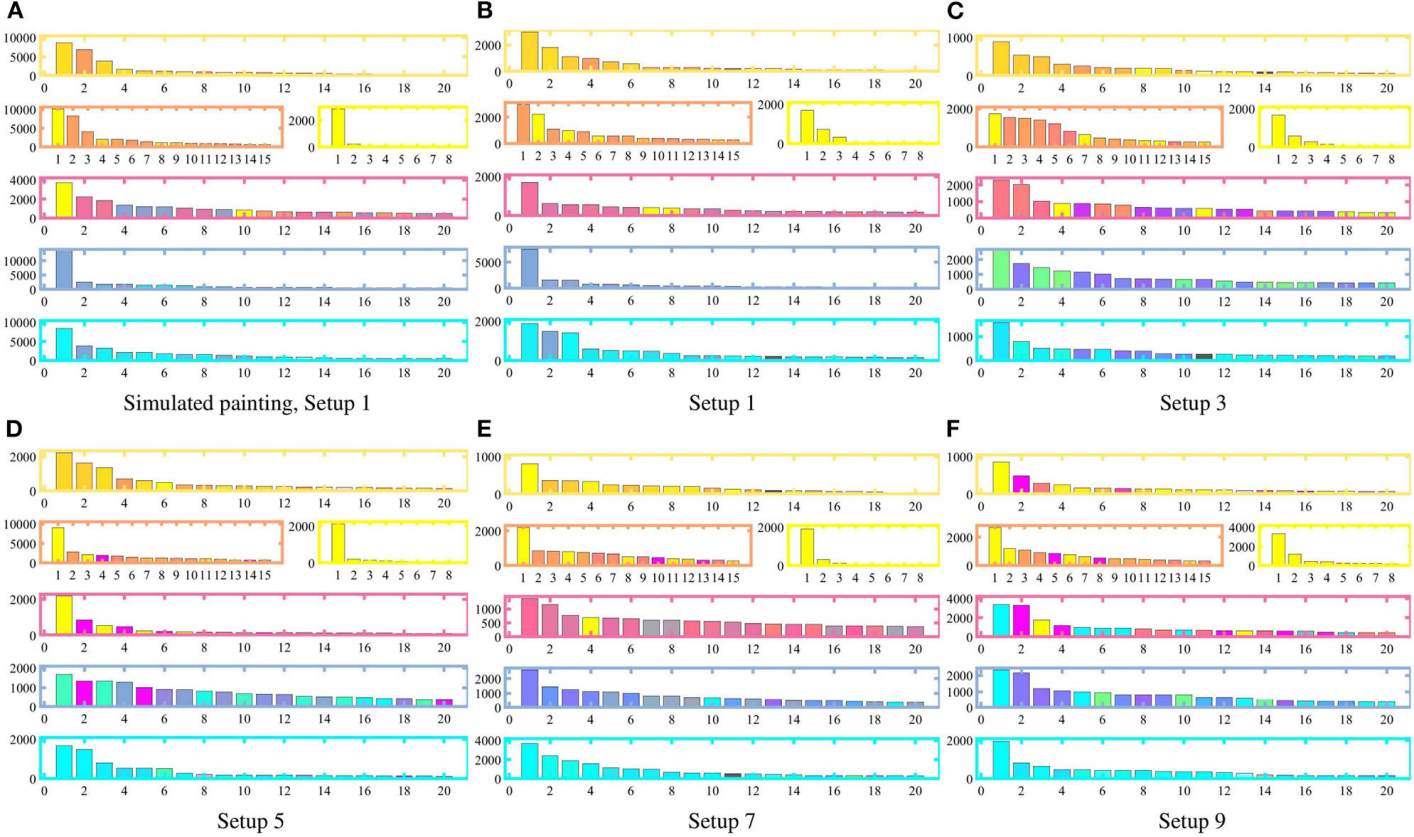
Chromospectroscopy by sectors on the canvas (as indicated in Table 3) for each equipment configuration (as specified in Table 2). With increasing heterogeneity, and consequently, sparser painting capabilities of the robots, colors distinctly different from the target colors begin to appear in each sector. For teams with lower heterogeneity (Setups 1 and 3), anomalous colors in the chromospectroscopy typically appear from neighboring sectors only.

The second method we utilize to quantify the differences among the paintings as a function of the heterogeneity in the robot team is using *chromospectroscopy* (Kim et al., 2014), which analyzes the frequency of occurrence of a particular color over the canvas. To this end, the painting is divided according to the sectors described in Table 3, which are closely related to the areas of high incidence of the objective color densities in Table 1. A histogram representing the frequency of occurrence of each input color per sector is described in Figure 8. For the purposes of the chromospectroscopy analysis, the 8-bit RGB color map of the canvas is converted into a 5-bit RGB color map, by reducing the resolution of the color map and grouping very similar colors together, i.e., for an input color β ∈ [0, 255]^3^, the modified color for the chromospectroscopy analysis in Figure 8 is computed as $\bar{\beta }=\frac{\beta }{b}$, with *b* = 2^3^.

**Table 3:** Color sectors throughout the painting used for the chromospectroscopy analysis, according to the density parameters specified in Table 1.

**Sector ID**	**Objective color**	**x_min_[m]**	**x_max_[m]**	**y_min_[m]**	**y_max_[m]**
1	Yellow	−1.2	1.2	0.6	1
2	Orange	−1.2	1.2	0.2	0.6
3	Pink	−1.2	1.2	0.2	0.2
4	Blue	−1.2	1.2	−0.6	−0.2
5	Cyan	−1.2	1.2	−1	−0.6
6	Yellow Sun	0.3	0.7	0.1	0.5

As seen in Figure 8, the heterogeneity of the robot team significantly affects the resulting color distribution within each sector. More specifically, as the heterogeneity of the team increases, thus depriving the team of painting capabilities, the canvas presents more outlier colors which are present outside the corresponding target sectors. This is apparent in highly heterogeneous teams (Setup 9), where magenta-like colors appear in the top-most sector and cyan appears in the central sector. The three central sectors show a high occurrence of non-target colors. For slightly lesser heterogeneous teams, while the occurring colors often do not correspond with the target colors in the sectors—e.g., green in Sector 4 of Setup 3—, the colors seem consistent in their presence and correspond to limitations on the equipment of the robots: in Setup 3, all robots are equipped with only two colors, thus no robot is able to exactly replicate any target color with 3 CMY components by itself. In the case of teams with low heterogeneity, e.g., Setup 1 and Setup 3, resulting colors are mostly consistent with the input target colors. The presence of some colors which do not match the input corresponds to colors belonging to the neighboring sectors. Some specific examples of this include: (i) Setup 1: the presence of yellow in Sector 3, orange in Sector 2, and Blue in Sector 5, (ii) Setup 3: the presence of orange in Sector 1, and blue in Sector 5, (iii) Setup 5: magenta and cyan-like colors in Sector 4.

Indeed, as one could expect, the chromospectroscopy reveals that color distributions become less precise as the differences in the painting capabilities of the robots become more acute—observable as distinct paint streaks in Figure 5 which stand out from the surrounding colors. Nevertheless, the distribution of colors on each sector still matches the color density inputs even for the case of highly heterogeneous teams, which suggests that the multi-robot painting paradigm presented in this paper is robust to limited painting capabilities on the multi-robot team due to restrictions on the available paints, payload limitations on the robotic platforms, or even the inherent resource depletion that may arise from the painting activity.

### 5.3. Statistical Results

**Figure 9:**
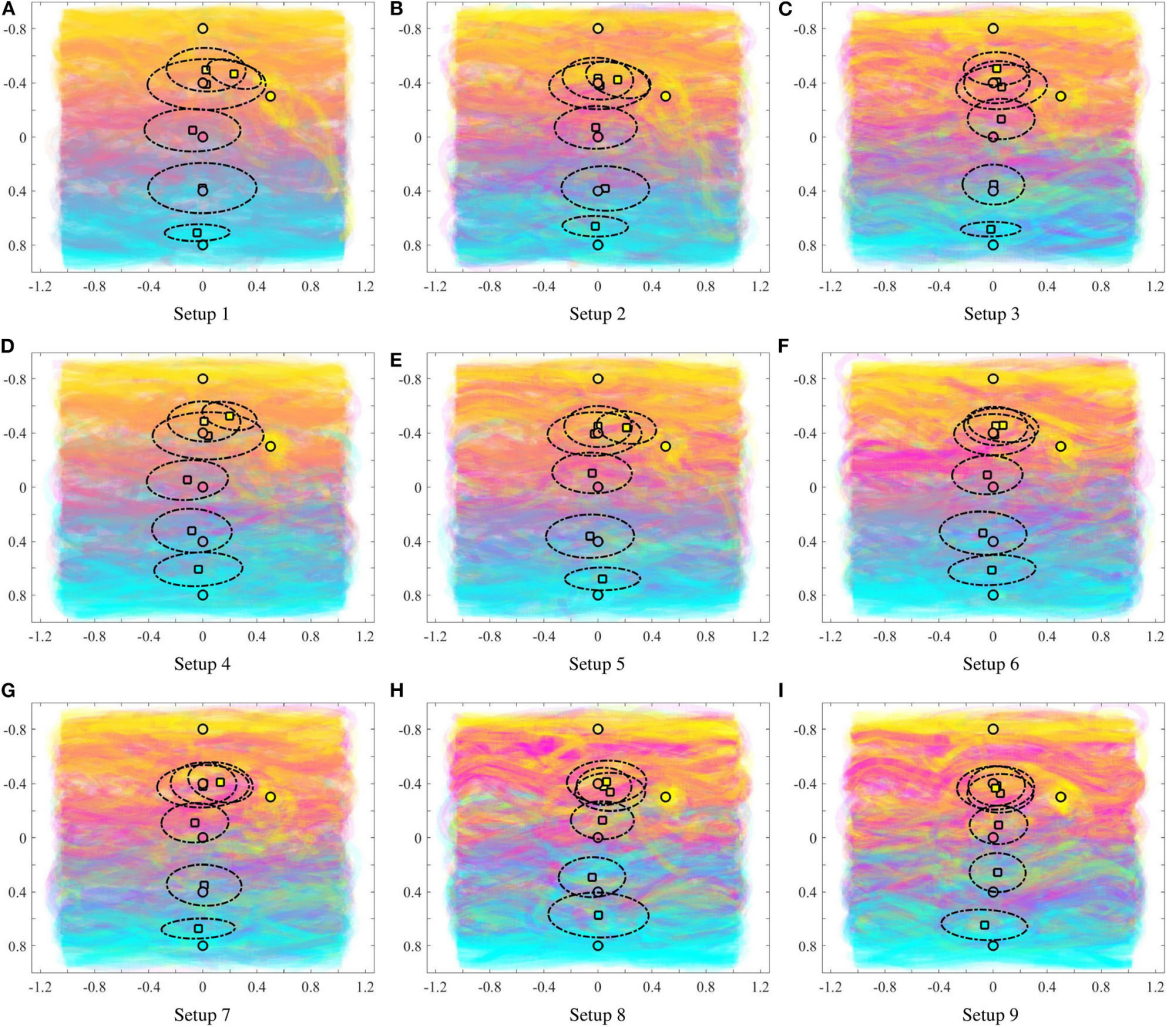
Averaged paintings over 10 trials. Mean of the input densities (circle), center of mass of the resulting colors according to φ from (13) (square), and covariance ellipse (dotted lines). The heterogeneity in the painting equipment of the robots has a significant impact on the nature of the paintings.

In order to understand if the statistics reported above remain consistent for multiple paintings generated by the robotic painting system, we ran 10 different experiments with random initial conditions in terms of robot poses for each of the 9 equipment configurations described in Table 2. Figure 9 shows the average of the paintings generated for each equipment, along with the color density averages, computed using (13). Although averaging the 10 rounds seems to dampen the presence of outliers, we can still observe how the distance between the objective color (represented by a circle) and the resulting color distribution (square) generally increases as the team becomes more heterogeneous. Furthermore, if we observe the color gradient along the vertical axis of the painting, the blending of the colors becomes more uneven as the heterogeneity of the team increases. This phenomenon becomes quite apparent if we compare the top row of Figures 9A–C to the bottom row (G–I).

**Figure 10:**
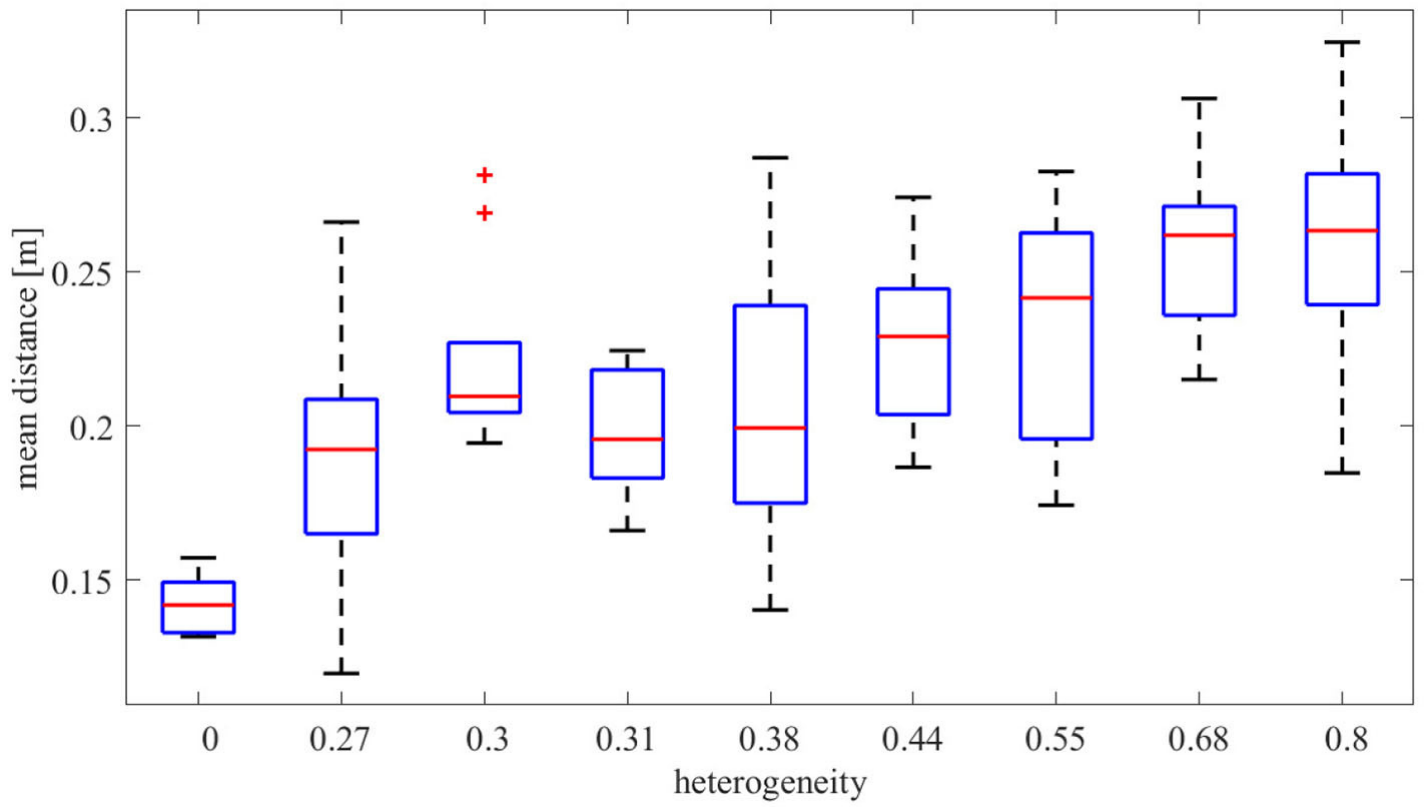
Box plots of the average distance between mean density input to resulting center of mass as computed in (15) for the 9 different equipment configurations. The results are presented for 10 different experiments conducted for each equipment. As seen, the average distance increases with increasing heterogeneity among the robots' painting equipment.

Quantitatively, this distancing between objective and obtained color density distribution is summarized in Figure 10, which shows the mean distance between the input density and the resulting colors. Analogously to the analysis in Figure 7, which contained data for one run in the Robotarium for five out of the nine setups, the average distances shown in Figure 10 show that the resulting color distributions tend to deviate from the objective ones as the team becomes more heterogeneous.

The results observed in this statistical analysis, thus, support the observations carried out in the analysis of the paintings obtained in the Robotarium. Therefore, the characterization of the painting outcome with respect to the resources of the team seems consistent throughout different runs and independent of the initial spatial conditions of the team.

## 6. Conclusions

This paper presents a robotic swarm painting system based on mobile robots leaving trails of paint as they move where a human user can influence the outcome of the painting by specifying desired color densities over the canvas. The interaction between the human user and the painting is enabled by means of a heterogeneous coverage paradigm where the robots distribute themselves over the domain according to the desired color outcomes and their painting capabilities, which may be limited. A color mixing strategy is proposed to allow each robot to adapt the color of its trail according to the color objectives specified by the user, within the painting capabilities of each robot. The proposed multi-robot painting system is evaluated experimentally to assess how the proposed color mixing strategy and the color equipments of the robots affect the resulting painted canvas. A series of experiments are run for a set of objective density functions, where the painting capabilities of the team are varied with the objective of studying how varying the painting equipment among the robots in the team affects the painting outcome. Analysis of the resulting paintings suggests that, while higher heterogeneity results in bigger deviations with respect to the user-specified density functions—as compared to homogeneous, i.e., fully equipped, teams—the paintings produced by the control strategy in this paper still achieve a distribution of color over the canvas that closely resembles the input even when the team has limited resources.

## Data Availability Statement

All datasets generated for this study are included in the article/Supplementary Material.

## Author Contributions

MS, GN, SM, and ME contributed to the conception and design of the study. MS, GN, and SM programmed the control of the robotic system and performed the design of experiments. MS conducted the data processing and the experiment analysis. MS, GN, and SM each wrote major sections of the manuscript. ME is the principal investigator associated with this project. All authors contributed to manuscript revision, read, and approved the submitted version.

## Conflict of Interest

The authors declare that the research was conducted in the absence of any commercial or financial relationships that could be construed as a potential conflict of interest.
